# X-ray perception: Animal studies of sensory and behavioral responses to X-rays

**DOI:** 10.3389/fncel.2022.917273

**Published:** 2022-08-02

**Authors:** Vaishnavi Mantraratnam, Jorge Bonnet, Caleb Rowe, Daniel Janko, Mark Bolding

**Affiliations:** ^1^Department of Radiology, University of Alabama at Birmingham, Birmingham, AL, United States; ^2^Department of Neurology, University of Alabama at Birmingham, Birmingham, AL, United States

**Keywords:** X-rays, optogenetics, ionizing radiation, sensory, vision

## Abstract

Since their discovery in 1895, many studies have been conducted to understand the effect of X-rays on neural function and behavior in animals. These studies examined a range of acute and chronic effects, and a subset of studies has attempted to determine if X-rays can produce any sensory responses. Here we review literature on animal behavioral responses to X-rays from 1895 until 2021 to assess the evidence for detection of X-rays by sensory receptors in animals. We focus on the changes in appetitive and consummatory behavior, radiotaxis, behavioral arousal, and olfactory responses to X-rays that have been reported in the literature. Taken together, the reviewed literature provides a large body of evidence that X-rays can induce sensory responses in a wide variety of animals and also suggests that these responses are mediated by known sensory receptors. Furthermore, we postulate the role of reactive oxygen species (ROS), the most biologically active byproduct of X-rays, as a key mediator of sensory receptor responses to X-rays.

## Introduction

X-rays have inspired fascination and curiosity since their discovery in 1895 by Wilhelm Conrad Röntgen and the effects of X-rays on animals and humans have been the focus of many investigations. The reaction to Röntgen's initial discovery was sensational and led to a veritable explosion of research on the mysterious rays and as early as 1897 when Freund had begun investigations into their biological effects (Widder, 2014).

X-rays are a form of high energy electromagnetic radiation that can penetrate matter more readily than visible light. At kilovolt energies (typical clinical values) the attenuation of X-rays per unit mass is approximately proportional to *Z*^3^/*E*^3^, where *Z* is the atomic number and E is the energy of the incident photon. As a result, X-rays are less attenuated by soft tissues and more attenuated by hard tissues and so X-rays can be used to produce the projections of tissue density known as radiographs. This property has led to the wide use of X-rays for clinical imaging. For higher Z materials (e.g., iodine, *Z* = 53), sharp increases in attenuation can be seen at energies near the binding energy of the inner shell electrons. These are known as k-edges and this property has led to the use of iodine and barium as clinical contrast agents. Abundant elements in tissue (i.e., carbon, hydrogen, oxygen, and nitrogen) have k-edges that are so low they are difficult to detect.

X-rays' impact on biology is principally by radiolysis of water. This is principally due to the ubiquity of water in biological systems. Essentially, a high energy photon can kick an electron out of a water molecule and into solution. This leads to a rapid cascade of events and the generation of reactive oxygen species (ROS) which have a wide range of reactivities. Importantly, at low concentrations ROS can act as a cellular signal and ROS plays a vital role in signal transduction, metabolic regulation, and homeostatic regulation in processes like apoptosis, autophagy, the cell cycle, and immunity (Moloney and Cotter, 2018; Unable to find information for 178238; Holmström and Finkel, 2014). At high concentrations ROS can damage a wide array of cellular components including DNA making ROS mutagenic at higher levels. Thus, X-rays could have significant modulatory effects on ROS-mediated signaling cascades and cellular function and these effects could vary dramatically with the dose delivered.

In current medical practice, X-ray dose is controlled and generally minimized to mitigate harmful effects while maintaining diagnostic utility (Tafti and Maani, 2022). As a result, much literature on X-ray perception derives from the earliest days of X-ray research when there was little awareness of their harmful effects. As awareness of these harmful effects has increased, research on the perceptual effects of X-rays has become rare. Investigations of the dose dependence of biological effects of X-rays is in some ways still not nuanced (Widder, 2014). Presently, a linear dose dependent harmful effect model is generally accepted though other models such as hormesis (i.e., low doses of ionizing radiation are beneficial) have good experimental support but limited acceptance (Baldwin and Grantham, 2015).

The objective of this review paper is to provide an overview of the many studies conducted to examine sensory effects or perception of X-rays by animals. In the current review we are concerned with the immediate sensory effects of X-rays rather than longer term biological effects of X-rays such as dermatitis, radiation sickness, or mutagenesis. This review does not include studies of the effects of X-rays on the production of lethal DNA mutations, or the directed evolution of X-rays resistance in bacteria or other prokaryotes. In particular, this review does not look at studies of the mutagenic and harmful effects of ionizing radiation in humans which have been the subject of other recent reviews (Rödel et al., 2017; Maqsudur Rashid et al., 2019; Shin et al., 2020). Finally, this review does not focus on studies of human perception of X-rays. While we may include studies on the perception of X-rays by humans in the chronology for context, they will not be discussed in depth. Human studies are the subject of a forthcoming review in preparation.

We are principally concerned with the questions of whether or not animals can have a sensory response to X-rays that is not simply due to tissue damage and, if so, what are the potential mechanisms driving this phenomenon. We discuss reports of specific sensory effects and suggest a common mechanism by which the sensory effects might arise. To facilitate discussion, we define specific meanings for several key concepts used in this paper. X-rays designate a penetrating form of high-energy electromagnetic radiation with wavelengths shorter than UV and longer than gamma rays. Here we define X-rays to have a wavelength ranging from 10 pm to 10 nm, corresponding to energies in the range 145 eV to 124 keV. As is consistent with most of the literature reviewed, we do not always make a clear distinction between X-rays and gamma rays. In this review “sensing,” “sensation,” and “perception” are inferred from behavioral or electrophysiological responses and can be considered synonymous unless otherwise noted. By “sensory receptor” we mean to indicate a membrane bound protein receptor such as a photo-, chemo-, or mechanoreceptor that is part of a sensory signaling pathway. At times “sensory receptor” may more broadly indicate the cell or organ that the protein receptor is a part of, and this should be clear from context. When the word “light” is used without qualification it indicates visible light and not ionizing radiation, UV, or infrared.

Prior to this paper, the most recent published review addressing questions in this area was Lipetz's review “The X-ray and radium phosphenes,” which concluded that the visual system can be stimulated by X-rays mediated by the photoreceptors of the retina, and that fluorescence of the ocular media is negligible and does not constitute a viable mechanism, except at very high intensities of X-rays (Lipetz, 1955). Since 1955, a body of X-ray perception literature has accumulated that has not yet been reviewed.

## Chronology

Röntgen is credited with the discovery of X-rays at the end of 1895. X-rays were initially described as invisible. By 1896, Brandes and Dorn reported that X-rays could produce phosphenes.^1^ This claim was initially met with some resistance by other investigators including Röntgen. However, by the end of 1897 there were numerous reports that X-rays could produce visual effects. There were investigations into the nature of X-ray phosphenes over the next few years and the site of action was determined to be the retina. However, by 1906 the interest in the phenomena seems to have been lost and eventually X-rays were again generally considered to be invisible.

In 1932 two investigators, Taft and Pirne independently rediscovered the visual effects of X-rays. This led to the use of X-rays for ophthalmology and for basic research into human vision. The visual effects of X-rays began to be used as clinical tools for the evaluation of visual perception and for research purposes over the next two decades. In 1955 Lipetz began the first of a two-part report on investigations into the mechanism of X-ray visual phenomena with the following paragraph.

“OPHTHALMOLOGISTS, as well as radiologists, are aware that radium radiations and X-rays can produce a sensation of light on striking a person's eye. This phenomenon is being used clinically to locate foreign bodies within the eye and to test the retinae of cataractous eyes. It has recently been used to measure the diameters and refractive power of living human eyes. But the mechanism by which these radiations arouse a visual sensation is still unknown.”

Around the time these two papers were published, several groups began investigations into the sensory effects of X-rays using a variety of animal models including monkeys, rats, insects, and crustaceans, which continued into the late 1970s. Kimeldorf was particularly active in this area and published papers over several decades and his book with Hunt entitled “Ionizing Radiation: Neural Function and Behavior” was published in 1965.

There were several particularly notable experiments over the era from the early 30s to the early 70s. Edward Baylor Frederick Smith conducted experiments on the perception of X-rays in Daphnia magnia (water fleas) and demonstrated phototaxis-like behavior in response to X-rays in 1958. Hunt, Garcia, and Kimmeldorf demonstrated radiation-induced conditioned avoidance and direct stimulation of the mammalian nervous system with X-rays in rats, mice, and cats in the early 1960s. These experiments were influenced by the first findings of sensory responses to X-rays shortly after the discovery of X-rays. In humans, multiple groups compared the properties of X-ray visual effects to the properties of light perception and deduced common properties and interactions between the perception of light and X-rays (Lipetz, 1955). X-rays perception was also used to measure the diameter of the globe by passing beams across the eye and having subjects report the percepts. Concurrently, X-ray perception was used clinically to look for foreign bodies in the eye by having patients report the location of the X-ray shadows (Godfrey et al., 1945).

As humans began to enter space, astronauts reported flashes of light during space travel. This led to space and terrestrial experiments on the visual effects of ionizing radiation in the 1970s and later in the 2010s. These experiments alongside the general interest in the effects of radiation prompted animal studies that sought to uncover the mechanisms of X-ray phosphenes throughout the 1950s into the 1970s (Doly et al., 1980a,b). Since the 1970s, there have been more sporadic but nonetheless highly informative experiments into the X-ray phosphene mechanism. In particular, Doly et al. (1980a,b) and Savchenko (1993) present convincing evidence that X-ray phosphenes in mammals are mediated by the rod cells of the retina. Since then, there have been multiple reports of human sensory perception of ionizing radiation during radiation therapies with cranial targets (Rödel et al., 2017; Maqsudur Rashid et al., 2019; Shin et al., 2020).

The history of reports of X-ray perception and significant developments is outlined in Table 1.

**Table 1:** A chronology of significant reports of X-ray perception.

**Year**	**Model**	**Notable report or time period**
1895	Human	Discovery of X-rays by Röntgen who states that X-rays are invisible.
**1895–1906**		The initial discoveries that X-rays can produce visual effects in humans and those animals exhibit phototaxis. The perception of X-rays by humans is found to require a dark-adapted vision—something that is found by all following investigators.
1896	Human and invertebrate	Brandes and Dorn report X-ray phosphenes, Axenfeld reports that insects and crustaceans exhibit phototaxis in response to X-rays and that this effect goes away if the animals are blinded (Lipetz, 1955).
1897	Human	Röntgen capitulates and reports that X-rays can produce visual responses (Lipetz, 1955).
1903	Human and vertebrate	Hardy and Anderson (1903) conclude that the site of X-ray phosphene production is the retina.
1906	Human	Except for reviews in 1910, 1912, and 1925, there are no more reports on X-ray phosphenes for many years. Lipetz (1955) speculates that this is because of increasing awareness of the harmful effects of X-rays.
**Early 1930s to late 1950s**		Visual effects of X-rays were used in clinical ophthalmology. “Seeing” lead letters and other targets with closed eyes is demonstrated. The psychophysics of X-ray “vision” investigated.
1932	Human	“Taft and Pirie independently rediscover the visibility of X-rays (Pirie, 1932; Taft, 1932). Pirie reports on identifying lead targets with closed eyes using X-rays (Pirie, 1932, 1934). Further reports were published about every 2 years until 1955 (Lipetz, 1955).
1941	Human	Newell and Borley (1941) measure the threshold X-ray intensity required to produce the phosphene in humans. The threshold varied from 0.5 to 1.4 r/min in three normal subjects for an area of 1 mm^2^. They found the time course of dark adaptation for X-rays and light to be similar.
1945	Human	Based on the method described by Pirie in 1934, Godfrey et al. (1945) describe a refined technique to locate foreign bodies in the eye or orbit by the X-ray shadows cast on the retina of a patient. Like Newell and Borley, they reported that the dark adaptation curves for light and X-rays were similar.
1951	Human	Lipetz (1955) reports that in several experiments Belluci found that the pupillary response to X-rays was consensual and that X-ray phosphenes exhibit persistence of vision. Belluci also found that phosphene brightness increased with tube voltage and current and the phosphene changed from blue to yellow green to yellow as current increases.
1953	Human	Bornschein et al. (1953) found the threshold of X-ray vision to vary from 1.6 to 8.7 mr/s and that the threshold dose was nearly constant for durations of stimulus <20 ms. For greater durations the threshold dose increased with stimulus duration
**Mid 1950s to Mid 1970s**		Multiple investigators experiment with the sensory effects of X-rays using a variety of animal models including monkeys, rats, insects, and crustaceans. Kimeldorf and his collaborators are particularly active in this area.
1955	Human	Lipetz reports that X-rays were being used clinically in 1955 to locate foreign bodies in the eye and to test the retinas of cataractous eyes, but the mechanism of X-ray perception was unknown (Lipetz, 1955)
1958	Invertebrate	Baylor and Smith (1958) report that water fleas demonstrate phototaxis in response to X-rays as they would to blue light.
1960	Vertebrate	Kimeldorf et al. (1960) report the demonstration of an avoidance behavior conditioned by radiation exposure. They state, “The stimulus tends to be unique in that a specific receptor system is not known.”
1960	Vertebrate	Garcia and Kimeldorf (1960) report that localized X-ray exposure of the head, thorax, abdomen, or pelvis served as a motivating stimulus to condition a saccharin aversion in rats.
1960s to 2000s	Human	Reports of light flashes from astronauts and other potential cosmic ray effects lead to ALFMED experiments during Apollo 16 and 17 transits in 1972 and the SilEye-Alteino and ALTEA projects aboard the MIR and ISS are performed in space 3 decades later. In both experiments astronauts wore helmets designed to capture the tracks of cosmic ray particles to determine if they coincided with the visual observation. It was concluded that the visual phenomena were caused by cosmic rays (Pinsky et al., 1975; Casolino et al., 2003a).
1960s	Vertebrate and invertebrate	In a series of papers, Bachofer and Wittry measure the Electroretinogram in response to X-ray stimulation in frogs and find that rhodopsin is sensitive to X-rays (Bachofer and Wittry, 1961, 1962; Bachofer and Esperance Wittry, 1963).
1962, 1963	Vertebrate	Hunt and Kimeldorf (1962) and Baldwin et al. (1963) report that rats can be aroused from sleep using X-rays and that this effect is not dependent on vision.
1962	Vertebrate	Barnes (1962) reports that behavioral avoidance of X-rays by rats can be eliminated by splanchnicectomy, but not ophthalmectomy.
1963	Vertebrate	Smith and Morris (1963) report conditioned avoidance to saccharine to low doses of X-rays and the response is minimally dulled with age.
1963	Vertebrate	Garcia and Buchwald (1963) report on successful use of X-rays as a conditioned stimulus in an operant conditioning experiment.
1963	Invertebrate	Smith et al. (1963) report that moths in a darkened room respond behaviorally to brief pulses of low dose X-rays.
1963	Invertebrate	Baldwin et al. (1963) find a light-like on-response to X-ray stimulation in cockroaches.
1964	Invertebrate	Smith and Kimeldorf (1964) report the electrophysiological responses of moth eyes to beta radiation and compare it to light stimulation. They conclude that the X-ray response elicits an ERG like that of a light response.
1964	Vertebrate	Garcia et al. (1964) report that rats can be trained to respond behaviorally to very low doses of X-rays and that the site of action is in or near the olfactory bulbs.
1965	Vertebrate	Feder (1965) and Feder et al. (1966) reports on perception of X-rays by rats and concludes that the response to low doses is due to olfactory bulbs and the avoidance response to high doses is abdominal.
1966	Vertebrate	Cooper and Kimeldorf (1966) report that rat olfactory bulb neurons respond to X-rays electrophysiologically by transiently and promptly increasing firing rate.
1970	Invertebrate	Kimmeldorf's student Jordan reports that ERG responses to X-rays in Purple shore crabs are similar to responses to light (Jordan, 1970). He obtains quantum efficiencies of about 1% which is in line with reports of Lipetz.
1970s	Human	In several experiments observers view neutron beams and other high energy radiation sources to try and determine the mechanism of cosmic ray induced light flashes. Two of the principal hypotheses are Cherenkov radiation and direct photoreceptor stimulation (Charman et al., 1971; Tobias et al., 1971).
1971	Invertebrate	Kimeldorf and Fortner (1971) report that sea anemone responds to X-ray stimulation with immediate tentacle withdrawal and oral disc closure.
1972	Vertebrate	Chaddock (1972) reports that X-ray stimulation varies as a function of different monochromatic background illumination in monkeys.
1972	Invertebrate	Martinsen and Kimeldorf (1972) report that carpenter ants can sense X-rays with antennal flagella.
1974	Invertebrate	Dedrick and Kimeldorf (1974) report that sea urchins can sense X-rays and it resembles phototaxis.
1975	Invertebrate	Kernek and Kimeldorf (1975) report that shrimp can sense X-rays. “Prompt arousal responses were characterized by vigorous motions of appendages and by advancing, rolling and re- treating movements”
1980	Vertebrate	In two papers Doly et al. (1980a,b) report on the use of electrophysiology and photochemistry approaches in a rodent model to investigate the mechanism of formation of X-ray phosphenes.
1993	Vertebrate	Savchenko (1993) shows evidence that X-ray phosphenes occur as a result of the excitation of the rod apparatus.
2003 to 2009	Human	Casolino et al. (2003b), Fuglesang et al. (2006), Sannita et al. (2006), Narici et al. (2009) review reports of visual phenomena in space and propose mechanisms.
**2010 to now**		The term X-genetics was coined by Rachel Barry and Ge Wang. Many reports of phosphenes and other sensory effects in humans during proton and X-ray therapy with cranial targets.
2015	Human	Wilhelm-Buchstab et al. (2015) report that phosphenes can be generated extra retinally by proton therapy.
2020	Human	Narici et al. (2020) report that many sensory illusions are invoked by proton therapy and that the sensations track the irradiation closely in time and the visual sensations are extra retinal.
2021	Vertebrate	Matsubara et al. (2021) report successful demonstrations of scintillator mediated X-genetics using macroscopic scintillators and light sensitive optogenetic receptors and Chen et al. (2021) demonstrate X-genetics using scintillating nanoparticles and light sensitive optogenetic receptors.

## Observations and experiments in animals

In this section, we discuss specific studies that were conducted to examine animal responses to X-ray. Animals and insects have been observed to exhibit behavioral responses to X-rays. Vertebrates tested include Mammalian models with rat, mouse, and monkey models. Invertebrates include ant, moth, cockroach, sea anemone, and crustacean models. Plants, fungi, and humans have also been studied and have been reported to have prompt electrophysiological, perceptual, and behavioral responses to low doses of X-rays. In this paper we are principally concerned with studies in animals. Though, for context, we have included human studies in the above chronology, in the sections below we limit our discussion to animal studies. Studies of human perception of X-rays will be reviewed in a forthcoming paper currently in preparation. Table 2 outlines studies over the most active period of research in this area.

**Table 2:** Significant findings from animal studies of X-ray perception from 1956 to 1993 by Period.

**Year**	**Overarching significant finding**	**Common name of animal model**	**Key papers**
1956	Rats exhibit decreased sugar water consumption despite being in a food and water deprived state when conditioned to associate drinking sugar water with X-ray exposure. Furthermore, X-rays are shown to disrupt gastrointestinal function.	Sprague-Dawley Rats	Garcia et al., 1956a,b
1958	Water fleas or Daphnia magna exhibit unique downward swimming patterns in aversion to X-rays through a process likely mediated by the nauplius eye.	Water fleas	Baylor and Smith, 1958
1960	X-ray conditioning behavior found to be mediated by the abdomen. The eyes, vagus nerve, adrenal glands, and pituitary glands are not involved in sensations seemingly triggered by gastric dysfunction. Association of a distinctive taste is generated by conditioning animals to associate X-rays with particular fluids. Cats, mice, and rats all exhibit this taste sensation, and no evidence indicates the sensation is painful. Rats exhibit X-ray avoidance by preferring shielded chambers over non-shielded chambered in presence of X-rays.	Sprague- Dawley Rats, mice, cats	Garcia and Kimeldorf, 1960; Kimeldorf et al., 1960
1962	The entire gastrointestinal tract is highly radiosensitive with mucosa in the duodenum being the first tissue to show effects to ionizing radiation. The damaging effects of X-rays are sensed by the breakdown of the mucosa of the duodenum, small intestines, and stomach, which later progresses to the mouth, esophagus, and rectum. Acute X-ray perception is mediated by reactions in the gastrointestinal tract that signal *via* the splanchnic nerves. Sensory mechanisms outside the abdomen cavity mediate avoidance behavior after the first 15 min of irradiation. Acute responses to X-rays can be abrogated by intraperitoneal injection of procaine or surgical excision of one or more splanchnic nerves that innervate the thoracic trunk in the abdomen abrogates X-ray avoidance.	Sprague-Dawley Rats	Barnes, 1962
1963	Age of rats does not impact X-ray perception. Moths respond to low-intensity X-rays. Threshold intensity to promptly awaken rats from sleep within seconds is 0.25 r/s, with EEG responses within 1 s at 0.2 r/s, and rats can be conditioned with stimuli as low as 0.001 r/s. Changes in X-ray intensity caused “on-off” responses in the eye of cockroaches. Mammalian neurons respond perceptually and adaptively to extremely low levels of X-rays *via* two separate X-ray perception phenomena. Firstly, EEG recordings across various mammal models support the “hit” theory whereby a fast-acting mechanism is mediated by the reticular activating system in response to higher X-ray intensities. Secondly, conditioning experiments in rats support a “hangover effect” theory whereby extremely low doses of X-rays cause avoidance behavior as a result of the accumulation of breakdown products stimulating gastric dysfunction as signaled by a peripheral afferent effect.	Sprague-Dawley Rats, Wistar rats, Cockroach	Baldwin et al., 1963; Garcia and Buchwald, 1963; Smith and Morris, 1963
1964	X-rays are believed to stimulate neurons *via* biochemical interactions with afferent signaling cascades in the brain and secondary effects are ruled out by citing how X-rays must directly be aligned with the olfactory bulb and its primary neurons. It is posited that X-rays disrupt a biochemical signaling cascade in the olfactory bulbs after experiments using a precise X-ray machine directed toward specific regions of the brain delivered from various angles reveal the olfactory bulbs to be extremely radiosensitive. Moths respond on ERG to beta-radiation and X-rays at 0.25 mr elicit flight activity.	Sprague-Dawley Rats, Moths	Hunt and Kimeldorf, 1964; Smith and Kimeldorf, 1964
1965	Rats' ability to sense 0.1 s 0.2 r/s burst of X-rays to avoid shock is abrogated by the removal of olfactory bulbs. Phosphenes are noted as requiring higher X-ray dosages (10 r/min) and X-ray stimulation enhances retinal sensitivity to light and lowers thresholds where phosphenes occur although no irreversible damage is noted. Cockroaches respond to 0.09 mr delivered at 5.2 r/min in a 1 ms pulse in a dark-adapted state and the migration of eye pigments related to dark adaptation is shown to enhance radiosensitivity. Cockroaches may respond to even smaller X-ray doses given better X-ray technologies.	Sprague-Dawley Rats	Baldwin and Sutherland, 1965; Feder, 1965
1966	“Ions produced by radiation… in the mucus surrounding the cilia of olfactory receptors… stimulate receptors.” X-rays cause activation and desynchronization of neurons in the olfactory bulb. Ablation of the olfactory bulb greatly diminished the impact of X-rays on sleeping rats. X-rays' impact on the olfactory bulb is dependent on normal sensory input. Alcohol washing of the nasal cavities in tracheostomized animals abrogated any influence of X-rays on neural activity, while saline washing temporarily abolished responses. Cooper recorded secondary olfactory neurons for these experiments. If Cooper used primary olfactory neurons, it could have refuted Kimdelorf's experiments using ozone, which reportedly ruled out that rats could “smell” X-rays. Rats respond to irradiation of the whole animal, head only, or olfactory bulbs and do not respond to the irradiation of the air surrounding the rats' nose, the body behind the head, or specifically the head posterior to the olfactory bulb.	Sprague-Dawley Rats	Cooper and Kimeldorf, 1966; Feder et al., 1966
1970-1971	Sea anemones detect X-rays precisely and quickly with immediate tentacle withdrawal and oral disc closure responses. ERG responses to X-rays in Purple shore crabs are similar to responses to light. Fluorescence may play a role.	Sea anemones	Jordan, 1970; Kimeldorf and Fortner, 1971
1972	Visual detection of X-rays by Rhesus monkey changes as a function of varied background illumination. Carpenter ants have rapid and precise behavioral responses. Antennal flagella's sensory receptors (olfactory or ocular) were important for X-ray detection.	Rhesus monkeys, carpenter ants	Chaddock, 1972; Martinsen and Kimeldorf, 1972
1974	Sea urchins can detect X-rays *via* a dermal light sense that involves photostimulation of dermal nerve cells.	Sea urchins	Dedrick and Kimeldorf, 1974
1975	Red Ghost shrimp have rapid arousal to X-rays characterized by fervent advancing, rolling, and retreating.	Red ghost shrimp	Kernek and Kimeldorf, 1975
1980	Rod cells of the retina underlie X-ray phosphenes rather than any other biologic component of the eye. X-rays efficiently bleach isolated rhodopsin, which induces action potentials as recorded by ERG. The irradiation of proteins, including rhodopsin, disrupts weaker bonds in proteins causing partial disorganization of conformations. Unlike visual light which is absorbed by the chromophoric 11-cis retinal of rhodopsin, the energy from X-rays is absorbed by the opsin disorganizing its spatial conformation to facilitate an energy transfer that frees retinal.	Albino Rats	Doly et al., 1980a,b
1993	X-ray phosphenes have two distinct component reactions that can be altered by sodium azide, sodium nitrate, monoiodoacetate and other substances as measured by ERG in frogs. ERG of X-ray phosphenes is declared an essential tool to parcellate the radiational excitability of the central nervous system, but no further publications investigate the phenomena.	Rana temporaria frogs	Savchenko, 1993

A variety of sources can be used to produce X-rays. The most commonly used source is an X-ray tube which consists of an anode and a cathode with a high potential difference (voltage) inside of an evacuated envelope that is usually made of glass. X-rays are produced when electrons strike the anode. The spectrum of X-ray emission is a function of the anode material and the voltage. A tube was used as the source in each of the experiments reviewed here. To a first approximation, the output of any X-ray tube is determined by the current, voltage, anode material, and spot size. The spot is the area of the anode from which the X-rays are emitted. X-ray intensity varies linearly with current. The radiation arriving at the irradiated sample is additionally affected by distance (intensity) and any intervening material such as the metal plates that are used to attenuate low energy X-rays (changes spectrum). The spectrum is determined by the voltage, anode material, and filtering. Intensity is determined by current but can vary in a non-trivial way with geometry. Knowing how these parameters vary over time should, in general, allow an approximation of the exposure of a sample on an axis with the beam to be made even if other details about the source are unknown. So, X-ray tubes are in some sense generic, though accurate estimates of intensity require measurements with calibrated detectors, because, for instance, intensity can vary with angle with respect to the beam axis in a way that differs from tube to tube. However, in the papers reviewed, there is wide variability in how the sources are described. Often key parameters or how they varied over time are not reported. Generally, an estimate of exposure or absorbed dose was reported along with some of the key parameters, but there is wide variability in the units used, how they were measured, and assumptions about absorption.

## Behavioral responses to various dosages of ionizing radiation

Behavioral responses have been observed from experiments with ionizing radiation and can be separated into three groups: (1) changes in food and water consumption, (2) radiotaxis (including flight in insects), attraction or avoidance of ionizing radiation and behavioral arousal (including measuring the heart rate, eye activity, wakefulness, oral dilation and tentacle retraction, movement of antennae), and (3) olfactory responses. This review focuses only on acute and immediate sensory or learning-related responses. We do not examine delayed responses other than avoidance or conditioning. Sensory responses are especially linked to the visual system in humans, however human visual perception of X-rays will be treated in a future review.

## Changes in food and water consumption in response to radiation

Acute changes in food and water consumption as a behavioral response were noted in at least six studies. Across these studies, ionizing radiation was used as a conditioning stimulus to manipulate various food and fluid preferences and intake. Animals learned to avoid behaviors associated with irradiation such as certain foods, rooms, and fluids.

**Figure 1:**
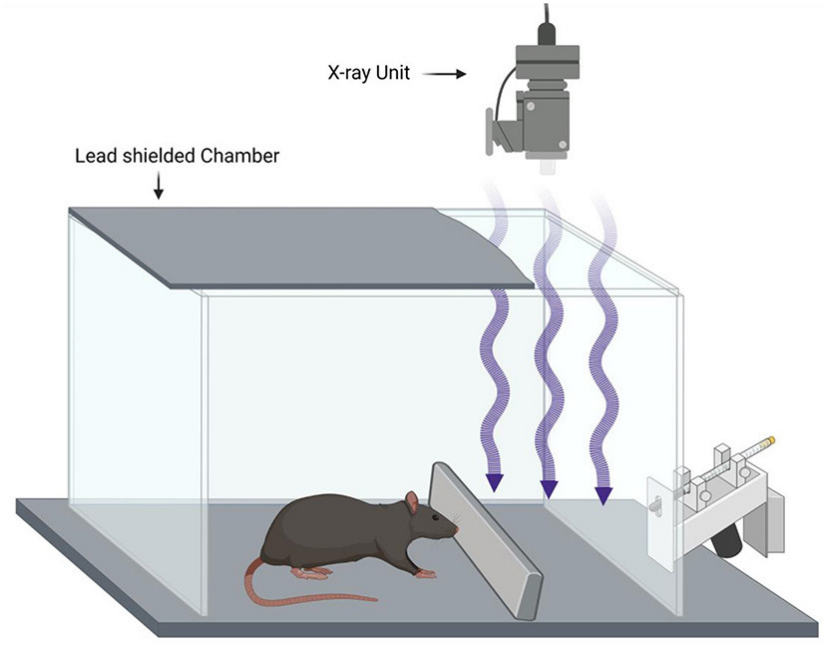
Example of a rodent X-ray conditioning experiment based on Feder (1965). Water restricted rats are conditioned to avoid a drinking tube by pairing X-ray exposure to a foot shock (unconditioned stimulus) providing evidence that the rats can make use of the X-ray exposure as a conditioned stimulus. Blocking the X-rays with lead prevents the effect showing that the X-rays are the conditioned stimulus and not some other cue.

In a 1965 article, a radiotherapy source was used with silent shutter devices according to the schematic shown in Figure 1.

Kimeldorf et al. (1960) conducted a study to see whether radiation induced a conditioned avoidance response in rats, mice, and cats, using a G.E. Maxitron deep-therapy unit to irradiate the three models of interest. The mean dose rate measured at the center of the exposure was around 0.66 R/min or ~0.011 R/s. All three species demonstrated a conditioned aversion toward radiation exposure and showed a progressive decrease in the amount of flavored fluid they consumed during each successive exposure. Garcia et al. (1956a) tested food and water consumption of rats surrounding 8 h exposures to gamma-radiation with ~7 curies of Co-60 contained in a brass capsule, which is equivalent to a radiation flux of ~9.4 R/h for a total dose amounting to ~75 R per exposure. In these studies, the rats' consumption of food and water decreased during each exposure to gamma-radiation for 8 h. There was a highly significant difference between the water consumption of sham-irradiated and irradiated animals (*p* < 0.001). Sham-irradiated animals gained weight; in contrast, irradiated animals lost several grams during each exposure to radiation. Water consumption was not significantly different after the first exposure, but after the eighth exposure, the water consumption of the rats increased for the entire period. Food intake of irradiated rats was not different from that of sham-irradiated controls after either exposure.

Garcia et al. (1956b) used the same Cobalt-60 source mentioned previously and exposed the animals for 8 h in a radiation field of 9.4 R/h. This again resulted in a total dose of 75 R. Food and water consumption decreased in groups which had multiple exposures earlier; controls who were subjected to sham-irradiation maintained their level of consumption or had increased consumption with repeated confinement.

Garcia and Kimeldorf (1960) used a G.E Maxiton X-ray machine to irradiate Sprague-Dawley rats at four radiation dose levels: 54 R, 108 R, 252 R, and 360 R and each dose level had 70 animals in the subdivision. Another group of 14 animals were exposed to a single 54 R whole-body irradiation. The resultant dose rate was around 1 R/min. The total dose was around 60 R for this group. A second group of hypophysectomized animals were exposed for 3 h to make a total dose of 180 R. The results from this study showed that the abdomen is a significant, critical area in establishing conditioned avoidance responses to ionizing radiation. Exposures of 54 R or 108 R delivered to the abdominal region led to a decrease in consumption. Similar doses had no considerable effect when the beam was directed to other areas of the body excluding the abdominal region.

In Smith and Morris (1963), an X-ray machine was used to irradiate Wistar rats for 20 min per day. X-ray dosages of 350 R, 300 R, 200 R, 150 R, 100 R, and 50 R were administered at a rate of around 30 R per min. This study found that the rats that had been conditioned to associate saccharin enriched water with being irradiated kept avoiding the saccharin water, independent of dosages above 50 R after being deprived of water.

Overall, these studies measuring the changes in food and water consumption in response to radiation suggest neural mechanisms relating to disturbances in the enteric nervous system. These hypotheses will be further explored in the “Proposed Neural Mechanisms” section below.

## Radiotaxis, avoidance, and behavioral arousal

Radiotaxis, a behavior analogous to phototaxis, and the more general behavior of avoidance of radiation is a response described in at least five studies from 1956 to 1993. Behavioral arousal is another reaction described in response to ionizing radiation. Responses that fall under this category include changes in heart rate, eye activity, wakefulness, oral dilation and tentacle retraction, as well as movement of antennae for specimens that do so. At least 12 studies between 1956 and 1993 described these reactions.

Baylor and Smith (1958) tested the perception of X-rays by daphnids with a type 200 B Kelley-Koet therapy unit. They exposed the animals to the X-ray for 30 min at a dose around 160–180 R/min. When irradiated by X-rays in the presence of red light, daphnids were found to swim downward as they do upon exposure to blue light, indicating a preferential stimulation of the nauplius eye.

Barnes (1962) tested peripheral neural paths mediating avoidance of radiation in rats using a Picker X-ray machine. A 40-min session of radiation of albino rats of the Sprague-Dawley strain was conducted. There were 1-min intervals applied during the first 15 min and then 5-min intervals for the remainder of the session. The unshielded compartment was irradiated around 32 R/min and the rate in a shielded compartment was around 0.75 R/min. The effect of this radiation was that the whole-body radiation led to avoidance behavior after 5–10 min of exposure in intact rats but not in rats with an intraperitoneal procaine injection or a splanchnicectomy, a surgical excision of a segment of one or more splanchnic nerves. The estimated dosage was around 16 R/min and until avoidance began, the rats spent only half of each in the compartment being irradiated at 32 R/min. They spent the remainder of each minute in the shielded compartment.

Hunt and Kimeldorf (1962) tested the stimulation of the nervous system with Sprague-Dawley rats using a Maxitron X-ray unit and exposed the rats to radiation for either 9 or 67 min. The dose rate for the rats in the high-intensity exposure group ranged between 1.5 and 2.5 R/s with a mean intensity around 1.9 R/s. The rate for the low-intensity exposure group ranged between 0.22 and 0.28 R/s with a mean intensity around 0.25 R/s. The high-intensity group displayed a significant peak in their heart rate at 30 s after exposure. The authors concluded that the threshold intensity of radiation to elicit clear neural activation and the diffusion of it (along with behavioral and heart responses) is around the range of 0.25–1.9 R/s. They also found that the threshold intensity for activation for behavioral arousal only is <0.25 R/s.

Smith et al. (1963) tested motor responses in moths to low-intensity X-ray exposure using a Westinghouse Mexitron X-ray machine. They irradiate the moths for 1–15 s with rest times between exposures ranging from several seconds to 3 min. The delivered dose rate was between 0.01 and 1.5 R/s. Their results suggested that the threshold intensity for the visual activation is almost equal to the intensity required to initiate wing beat in the moth. The start of flight activity may be a result of visual stimulation with low intensity radiation.

Garcia and Buchwald (1963) used an HVL X-ray machine to irradiate Sprague-Dawley rats for 10 s at the rate of 1.0 R/s and for 15 s at the rate of 0.2 R/s. They found that the rats can sense the X-ray at dose rates as low as 0.050 R/s when the X-ray is used as a conditioned stimulus; behavioral arousal was measured when the dose rate was as low as 0.2 R/s in this study as well.

Baldwin et al. (1963) found that cockroaches reacted to changes in light intensity with a typical on-off response, after operating with an X-ray machine at a dose rate of 2,000 R/min. The cockroaches displayed recovery after 30 s after the end of X-ray exposure in duration of 5 min. The experiment had short rest periods of 5 min after irradiation and the cockroach electrical activity displayed an amplitude decrease with small doses of around 300 R. Essentially, the large doses of radiation used in this study led to a decreased amplitude and frequency in the electrical activity in the cockroach eye.

Smith and Kimeldorf (1964) experimented with moths using beta radiation with three sources of Tracerlab Medical Applicators with 22-, 50-, and 87-Me of Strontium-90 in equilibrium with yttrium-90. They alternated with 1–2 s duration of flickering light and 87-mc beta radiation that consisted of eight stimuli per second. They measured the amplitude and response at 1, 2, 5, 10, 30, and 45 min. They found that the best dose rate threshold to see a response to the flickering train of beta radiation was around the range of 0.02–0.06 R/s and that the maximum amplitude response occurred within 5 min.

Hunt and Kimeldorf (1964) when experimenting with Sprague-Dawley rats with a Maxitron X-ray unit found signs of behavioral arousal and neural activation. The experiment consisted of a 2-h test period with 5-min intervals for the first 30 and 10-min intervals for the remainder of the 2-h period. The total dose was around 1,000 R delivered at a rate of 1.9 R/s for the high-dose group and 0.25 R/s for the low-dose group. They noted arousal spikes during sleep, faster heart rate, and more wakefulness as measured by “active” ratings.

Baldwin and Sutherland (1965) experimented with the Cockroach Blaberus with an X-ray machine and found that in the eye, the smallest total dose that produced an electrical response was 0.09 mR delivered at a dose rate of 5.2 R/min for a pulse of 1-ms duration that created an actual dose rate below 1.5 R/s.

Jordan (1970) tested on the purple shore crab *Hemigrapsus nudus* with a General Electric model D-2 diagnostic X-ray machine and subjected the specimens to two flashes per second or constant exposure. The minimum beta dose that was created that led to a measurable visual “on” response was around 0.9 to 1.6 mR. The “on” responses consisted of visual responses and this study indicated that the structure of the eye and fluorescence may shape the “action spectrum.”

Kimeldorf and Fortner (1971) tested the detection of ionizing radiations by a marine coelenterate, sea anemones, that were irradiated for 4 days at a maximum dosage of 20 R/s. The reaction time of the sea anemones ranged from 102 s at 20 R/s to 266 s at 1 R/s. At these times, tentacle withdrawal was observed. As the amount of exposure increased, the sea anemones further reacted, and the depressing of their peristome was observed with the dilation of the stomodeum. Furthermore, the oral disc was closed entirely as the extreme type of reaction, which is the strongest defensive reaction that the sea anemone can produce in response to noxious stimuli. For the tentacle retraction, reaction times ranged from 100 s at 20 R/s to 201 s at 2 R/s and other responses, including oral disk closure and mouth dilation, required much longer reaction times.

Martinsen and Kimeldorf (1972) tested the prompt detection of ionizing radiation by carpenter ants with a general electric Maxitron 300 therapy unit to irradiate the ants for 30 s at exposure rate below 0.7 R/s and up to 80 R/s. Fast behavioral responses were found to occur at rates around 0.05 R/s, including prompt, reflex-like responses such as head bobbing, brisk waving of the antennae, and rapid running behavior. This study also found that excitable tissues were stimulated by something inherent within them or accompanying exposure to X-rays. Carpenter ants also exhibited behavioral responses to radiation at 10 R/s within 1 s after the start of exposure. Overall, this study showed that in the range of 0.05–80 R/s, the strength and longevity of the responses was proportional to the exposure rate. The delay of the behavioral response was inversely related to the exposure rate.

Chaddock (1972) used a Universal X-ray machine with a rate of around 2–3 mR/s to irradiate Rhesus monkeys. The experiment used 15 s of targeted X-ray exposure to the head exposure to X-rays after which electric shocks were delivered for 0–3 s (Chaddock, 1972). The results of this experiment indicate that the sensitivity of the monkeys to detect X-ray stimulation depends on the monochromatic background illumination. The sensitivity of the monkey steadily decreased as the background illumination changed from red to blue at all exposure rates. Therefore, red was the most sensitive background illumination and blue was the least sensitive.

Dedrick and Kimeldorf (1974) tested the effects of ionizing radiation on sea urchins using a General Electric Maxitron-300 therapy unit. The duration of the exposure was 1–6 s and the dose rates ranged between 1 and 15 R/s. Instantaneous behavioral effects to X-ray exposure were seen; the reaction times were “related inversely to the exposure rate in the manner of a neurogenic stimulus.”

Kernek and Kimeldorf (1975) experimented with Red Ghost Shrimp, *Callianassa californiensis*, with GE Maxitron X-ray machine for an X-ray exposure for 150 s. This was a behavioral study in which five experiments were conducted using a 52 R/s dose rate and 1 using a 10 R/s dose rate. The responses measured from this study indicate that the X-ray serves as an excitatory stimulus and causes the shrimp to have fast arousal responses including the “motions of appendages” and grooming activity.

Doly et al. (1980a,b) conducted two studies on the retina of albino rats. In the first study, they used X-ray stimulation on the retina of albino rats. They used the X-ray Philips-11409 machine with a measured dose rate of 100 kV and 100 mA while delivering 80-ms pulses. The albino rats were under adaptation to darkness for 4–5 h, before the start of the experiment. The rats were killed and one of the eyes was removed to perform the isolation of the retina to then put it in a petri dish filled with a perfusion medium. The stimulation to the isolated retina was conducted in a dark room with a dim red light (wavelength > 610 nm). Under these conditions the isolated retina remained feasible for 4–6 h. Doly and the others postulated that it is possible to measure an electroretinogram from a “mammalian-isolated” retina that is exposed to X-rays. In addition, the electrophysiological response was due to the functionality and activity of the photoreceptors and the rods in rats because the light-adapted retina was not stimulated by radiation. With such conclusions, Doly and the others in the second study performed X-ray irradiation of rhodopsin extracts of the albino rats through the use of a RT-50 Massiot-Phillips X-ray tube for contact radiotherapy with a measured dose rate of 50 kV and 2 mA. They observed that the stimulation of photoreceptor tissue was caused by the X-rays using ERG (electroretinogram). They postulated that X-rays act on the rhodopsin and that ERG on isolated retinas demonstrated bleaching of the photopigments by X-rays. The retina's comparatively high radiosensitivity compared to isolated nerve fibers lead Doly and the others to believe that X rays act on the rhodopsin.

Savchenko (1993) worked on *Rana temporaria* frogs and used an X-ray diagnostic apparatus and subjected the frogs to stimuli at intervals of 2 min for 30–60 min. The dosage rates given were 3, 8, 16, and 53 R/s. This study concluded that the X-ray phosphene occurred due to the excitation of the rod cells, after oscillatory potentials were found in response to radiation and it was inferred that this excitement occurred in retinal cells, including the cell types horizontal, amacrine, or glial.

A recent study conducted by Lima and others in 2021 does not measure dose rate in R/s but instead in units of grays, which is equivalent to 100 rads. Lima et al. (2021) tested cortical electrical activity in water-deprived Wistar rats after exposure from a Clinac 600 C linear accelerator to visualize the effect of supplementation with omega-3. The rats were exposed for 5.02 min, and the dose applied was 2.4 Gy and in total 18 Gy of X-ray radiation was applied. 9 Gy was on the top of the head and another 9 Gy was applied on the bottom of the head of the rats. Radiation led to an increase in theta rhythm, regardless of omega-3 given or not.

## Olfactory responses

Olfactory responses are another reaction observed as a response to ionizing radiation. Responses that fall under this category include olfactory bulb activation.

A study by Feder (1965) exposed Sprague-Dawley rats to X-ray radiation for 0.1s at a rate of 200 mR/s. The significant findings related to olfactory responses from this study were that there was a drastic decrease in the ability of the rat to detect radiation after its olfactory bulbs were destroyed or the nostril was flooded with alcohol. Cooper and Kimeldorf (1966) also used Sprague-Dawley rats and tested the effect of X-rays on the rat's olfactory bulb. In this experiment, a Westinghouse X-ray unit was used, and the animals were exposed to the radiation for 1–5 s. The dose rates ranged between 1.5 and 2.0 R/s, but in some cases were as low as 0.05 R/s. They found that olfactory bulb neurons do respond to X-rays but were not able to make any significant conclusions about the different types of olfactory responses. The strength of the olfactory response in this study indicated that the dose rates of 1.5–2.0 R/s are above the threshold for sensing X-rays. The conclusions from this study were that radiation on the posterior region of the head or body is limited and is inadequate in changing the activity of olfactory bulb neurons.

## Proposed neural mechanisms

Neural mechanisms have been investigated in animal studies involving measuring the response of animal sensation and perception after the use of X-rays. From these studies, we deduce three main neural mechanisms by which animals may perceive X-rays: visual perception, signal cascade disruption, and olfactory perception. In vision, the retina mainly underlies this perception (Mathis et al., 2017). In olfaction, distinct reactive oxygen species (ROS) related modifications to the pre-existing environment are likely being sensed unless a sensory protein exists (Cooper and Kimeldorf, 1966). In signal cascade disruption, ROS likely modifies proteins or signaling molecules, acts as a signaling molecule, or perturbs redox balance. Past studies have concluded that radiolysis of water predominates X-rays impact on biological systems leading to the generation of ROS (Zaider). Specifically, pH-neutral aqueous solutions produce ~42 nM diatomic hydrogens, ~60 nM hydrogen atoms, ~71 nM hydrogen peroxide, ~222 nM hydroxyl radicals, ~230 nM solvated electrons per Gy of energy deposited (Spinks and Woods, 1990). ROS is a well-documented 2nd messenger signal, but a comparably vital component of radiosensitivity is tryptophan residues (Davies, 2003). Interestingly, tryptophan residues readily participate in redox chemistry when electron-rich; react rapidly with radiogenic hydroxyl radicals, hydrated electrons, and hydrogen atoms; act as an photosensitizers in UV photoabsorption to generate ROS; and are oxidized by UV (Ehrenshaft et al., 2015). Thus, tryptophan residue interactions likely mediate a biochemical mechanism of radiosensitivity. Across all these mechanisms, ROS and tryptophan residues play a pivotal role in the radiosensitivity of biological systems.

The potential that X-rays mediated behavior derives from direct interaction of X-ray photons with sensory receptors is almost impossible. X-ray absorption is primarily determined by atomic number and since specific tryptophan residues in radiosensitive proteins underlie avoidance behaviors in animals like *C. elegans*, the X-rays certainly operate *via* a chemical intermediate (Gong et al., 2016). Any specialized X-ray detection conformation is improbable. Our lab has calculated that for a “~50 kDa protein… only about one out of 50 million molecules… can be expected to absorb an X-ray photon per Gy of irradiation” (Cannon et al., 2019). In other words, it is highly unlikely that individual high-energy photons are interacting with specialized “X-ray sensitive” protein conformations to initiate a signal transduction mechanism. The anomalistic absorbed X-ray photon (the one out of 50 million for a ~50 kDa protein) would have to be absorbed at the proper region and orientation to cause a conformational change if this hypothesis were true. Such a conformation likely does not exist. Instead, X-ray perception is likely mediated by the most biochemically prevalent byproduct of X-rays passing through a system, ROS, which conveniently is a well-established regulator of various ubiquitous signal transduction pathways. Furthermore, from an evolutionary perspective, if an animal relied on any specialized protein conformation for sensing specific X-ray collisions as indicative to harmful radiation, why would biology evolve to select for highly unlikely collisions with source photons rather than the most ubiquitously active molecules emitting from those source photons? In other words, animals relying on a specialized protein conformation for sensing specific X-ray collisions would be vastly outperformed by animals able to sense the more ubiquitous chemical byproducts of harmful radiation. Biology favors the biochemical evolution of functional proteins and proteins that sense redox balance would have robust applications. Such proteins are known to exist across the animal kingdom despite our limited ability to measure radical chemistry in biological systems (Sies and Jones, 2020). Ultimately, direct photosensing proteins with specialized “single-collision-event” conformations are highly unlikely. A forthcoming paper will discuss mechanisms of X-ray perception more holistically by incorporating a broader range of biology.

## Visual perception

Visual perception or sensation of X-rays is a common and well-documented phenomena in humans. Studies of visual perception in humans will be reviewed in a forthcoming paper. In animal studies, photoreceptors and retinas have been implicated in X-ray perception in both vertebrates and invertebrates. The following section is organized chronologically.

In a 1970 study by Jordan Nelmichael, *Hemigrapsus nudus*, or purple shore crabs, were exposed to ionizing and non-ionizing radiation to explore the detection of visual responses (Jordan, 1970). Such bioelectric responses from the compound eye of the purple shore crab have been measured using an electroretinogram with light stimulation. The peak sensitivities to the stimulation of light were a result of each of the light absorption traits of rhodopsin, and fluorescence of the eye systems of the crab when ultraviolet light was used. These effects were taken into consideration because of the direct stimulation of the photopigment and possible secondary stimulation produced by fluorescence. The X-irradiation produced the same electrophysiological responses seen with the visible light at the start and end of the stimulation. Due to the low photon efficiency of this radiation, a direct influence on the photoreceptor mechanism was deemed unlikely.

Surprisingly, Kimeldorf and Fortner (1971) recorded behavioral responses to X-rays in Anthopleura xanthogrammica, a sea anemone, as well. This discovery further implicates X-ray perception across the animal kingdom. When irradiated, the sea anemone showed responses such as oral disc closure and tentacle retraction. Increased exposure resulted in faster tentacle retraction and immediate oral disc closure as a defensive reaction. Kimeldorf and Fortner suggested that a photoreceptor stimulation initiated by a wide spectrum of photon energies (of which need to be classified) were being activated. Another study co-authored by Kimeldorf investigated the immediate behavioral responses of the echinoderms *Strongylocentrotus purpuratus*, or sea urchin, to ionizing radiation (Dedrick and Kimeldorf, 1974). The findings showed that the sensitivity and efficiency of invertebrate's radiation detection improves as sense organs grow, particularly vision and olfaction. Although sea urchins lack visual organs, they have light sensitivity meditated by a dermal light sense. The dermal light sense involves nerve discharges that come from photostimulation of dermal cells. A 1-min exposure to strong light significantly increased the time it took for spines to react to X-rays. Thus, X-rays do not directly affect the subdermal nerve net, but rather excite photoreceptors.

Savchenko (1993) conducted a study recording electroretinogram responses to X-rays in male *Rana temporaria* frogs. The radiation stimulated excitation in the rod cells and X-ray responses were recorded *via* this excitation. Savchenko reported that it was not necessary to isomerize the photopigment. He found that any process that leads to the “piercing” of the membrane by a radical can set off events that change the cell's excitability, and this could be observed in the retina due to molecular amplification of the signal causing widespread excitation in the frog retina. He conjectured that these X-ray-specific retinal reactions could be used to test the responsiveness of the central nervous system through an X-ray phosphene mechanism. Savchenko concluded that there is a signaling cascade disruption caused by radiation resulting in a visual response.

Overall, photoreceptor systems are implicated in X-ray perception, but more studies are needed to identify mechanisms and receptors that underpin these reactions. Specifically, visual X-ray perception studies have lacked molecular interventions that would delineate whether disrupted phototransduction or direct photoactivation is occurring.

## Signaling disruption

The amplifying mechanism suggested by Savchenko is another possible neural mechanism for X-ray perception. Several studies expand upon and conjecture how X-ray irradiation leads to the disruption or amplification of neural signaling. This section is organized chronologically.

Garcia et al. (1956b) noted how Sprague-Dawley rats could be conditioned to alter food and water consumption using various levels of gamma radiation. They suggested that radiation impacted cholinergic nerves in the intestine which induced “an emotional state… reflected by… decrease[d]… consummatory behavior.” Years later, the same group discovered that the abdomen was most sensitive to irradiation aversion of all the body parts they irradiated individually (Kimeldorf et al., 1960; Hunt and Kimeldorf, 1962). Nevertheless, head, thorax, abdomen, or pelvis were all sensitive, but none as sensitive as full body irradiation leading them to hypothesize stomach dysfunction underlies radiation's control on behavior. Their hypothesis fails to account for radiosensitivity of organs beyond the stomach. Experiments irradiating the head of opthalmectomized rats would rule out a neural mechanism in the skull, while irradiating the extremities would rule out peripheral nerve responses. Furthermore, X-rays interaction with the phototransduction had not been explored at this point although they knew that the eyes did react. Kimeldorf et al. (1960) conducted a study on the radiation-induced conditioned avoidance behavior in rats, mice, and cats. From these studies, they deduced that the eyes respond to X-rays in a more sensitive dose-dependent manner, than the rest of the body—though they still had not identified any mediators or mechanistic factors for these phenomena. Kimeldorf and Hunt addressed neural responses in the mammalian nervous system, adding that the extent of this enteric neural response was determined by radiation intensity. Furthermore, they suggest that arousal was not dependent upon direct visual stimulation by X-rays noting arousal occurred and persisted after irradiation, implicating a reflex activation of the adrenal medulla. This adrenal response could be directly related to the effects of X-rays or caused by a fear response to the perception of X-rays. Irradiation localized specifically to the head and adrenal medulla separately while measuring adrenal stimulation would rule out whether a direct interaction is occurring or not. In the same year, Barnes (1962) published a study a month before Kimeldorf and Hunt and found that Albino rats of the Sprague-Dawley strain displayed avoidance after radiation. Barnes implicated an early enteric neural response caused by a disturbance of gastric and intestinal mucosa, beginning in duodenum eliciting avoidance behavior.

Garcia and Buchwald (1963) conducted a study using Sprague-Dawley rats to analyze the perception of ionizing radiation by studying behavioral and electrical responses to very low doses of X-rays. They emphasized that they didn't agree with Hunt and Kimeldorf that the behavioral arousal from X-rays is evidence for “direct central neural effects”; instead, they indicate a “peripheral afferent effect” is occurring. They bring up that EEG desynchronization and behavioral arousal does not “constitute evidence for direct effects upon the central nervous system.” They instead indicate that (1) there may be a detection mechanism similar to other sensory modalities that leads to an arousal response through the activation of the brainstem reticular formation or (2) there may be another response system with a lower threshold needed that depends on the total dose of radiation and time in order to create an effect. This disagreement with the previous study from Hunt and Kimeldorf (1962) is fascinating; though their data agreed with Hunt and Kimeldorf, they indicate that their hypotheses are different and diverge into indications that there is a peripheral effect instead of a central nervous system effect in the adrenal medulla.

Furthermore, Hunt and Kimeldorf (1964) in another study analyzing behavioral arousal and neural activation as radiosensitive reactions argue that the heart rate and arousal reactions to X-ray exposure in Sprague-Dawley rats tends to reject the idea of stimulation *via* abscopal effects at sites that are away from nervous tissue but agrees with the concept that ionizing radiation can stimulate the nervous system. In this study, they argued that the sensory deprivation could be caused by cortical inhibition and that chemoreceptors are most likely stimulated by ionizing radiation, since they have radiosensitive biochemical systems at the transduction or early amplification stages of receptor function. They suggested future studies of the radiosensitivity of chemoreceptors. They also postulate that penetrating ionizing radiation acts as a stimulus by causing energy transfer with irradiation through large areas of nervous tissue. They state that the effectiveness of radiation as a “distributed stimulus” depends on the “differential density of sensitive structures, the functional organization of neural elements, and the momentary state of excitability of those portions of the nervous system that are exposed.” Kimeldorf then collaborated with Smith, testing the bioelectrical response of the moth eye to beta-radiation, and found in this study that it is not clear if the beta radiation, the stimulus, acts on the receptor or if the effects seen in the moth eye are a secondary effect from fluorescence caused by irradiation (Smith and Kimeldorf, 1964). These two studies use different animal models, and it may be hard to reconcile these two studies. Though receptors and the activity of them can be generalized due to shared receptor functions, contrasting different mechanisms for cascade signaling may be important between vertebrates and invertebrates.

The *Callianassa californiensis*, known as the red ghost shrimp, was used in a study by Kernek and Kimeldorf (1975) for the discovery of behavioral responses and neural mechanisms with the use of X-rays. The removal of the antennules and limitations to the abdomen's exposure to the X-rays did not depress the shrimp's avoidance behavioral response, which indicated the activation of a complex and well distributed chemoreceptor system. The responses from the antennule and swimmeret preparations were very similar and that indicated that there was a common type of chemoreceptor reacting in both sites. Such chemoreceptors were located in all of the macrurus decapod's appendages. Due to the distribution of these chemoreceptors in the red ghost shrimp being studied, the critical receptor was indeed a type of chemoreceptor and the reason for the behavioral responses behind its neural signaling pathway.

There is a clear pattern of non-photoreceptor neural signaling effects identified in these studies in response to ionizing radiation. Future studies are needed to identify specific affected neurons in the central or peripheral nervous system and test the radiosensitivity of receptor types—especially chemoreceptors—and identify how the radiation acts on the receptors themselves.

## Olfactory perception

Olfactory perception of X-rays has been observed in animals with well-developed olfaction. In these studies, the neural mechanisms are specifically concerned with the olfactory bulb. This section is organized chronologically.

Cooper and Kimeldorf (1966) examined the activity of neurons in a rat's olfactory bulb in response to X-rays; they illustrated that olfactory bulb neurons do respond when exposed to irradiation through the use of the advancement of a microelectrode going through the olfactory bulb until a unit or response had been obtained with an amplitude that remained stable over a period of at least 5 min. The limitation in the study was that the rate and pattern of unit activity in the olfactory bulb varies greatly from unit to unit under resting conditions, as do the rat's responses to odors, even though several types of responses of olfactory bulb units to X-irradiation were described. Furthermore, the strength of the responses of many units to irradiation indicate that the dose rates that were used in this study are considerably above threshold. There were no “systematic threshold determinations” made in this study, but some units were studied which responded to the accumulated dosages of 20 mR or less delivered at a dose rate of 50 mR/s. The conclusion provided by the authors was that; “We can only conclude at present, therefore, that radiation impinging on the posterior part of the head or on the body only is of inappropriate quality of insufficient intensity [at the dose rates used in this study] to alter the activity of olfactory bulb neuron.”

Feder et al. tested the detection of minute doses of ionizing radiation in Sprague-Dawley rats (Feder, 1965; Feder et al., 1966). In the experiments, the opthamlactomized rat learned that the presence of the radiation will come before an electric shock to the paw, indicating the radiation was a cue for the shock. The researchers concluded that a receptor-like mechanism for the detection of the X-rays in the rat is centered in or around the olfactory bulbs. One shortcoming of this study is that their conclusion is not adequately supported; they should have irradiated the olfactory bulb and based their conclusion from that. Another approach to address this shortcoming is to irradiate the olfactory receptors in the nasal area of Sprague-Dawley rats. Perhaps, there is a possibility of detecting X-rays at the peripheral level of the nervous system, at the nose where olfactory receptor neurons are located.

Martinsen and Kimeldorf (1972) used carpenter ants to analyze their prompt detection of ionizing radiation. They suggested that sensory receptors at the antennal flagella had been often answerable for detection and were the cause of the onset of “off” responses, as ants with shellacked antennae did show detection after exposure, suggesting that the effect of the X-rays was centered around or on the antennal receptor.

There is clearly a pattern of signaling cascades within olfactory perception that become impacted by ionizing radiation. In the future, it will be important to identify the regions implicated in signaling cascades in the olfactory bulb area and receptors involved in olfaction.

## Discussion and future experiments

Based on reports over several decades, it is evident that animals can sense or perceive X-rays. X-rays were shown to induce neural activity changes, conditioned behavioral responses, and prompt behavioral responses in animals. This is supported by a large number of studies showing rapid behavioral responses that are neuronally mediated and have sensory-like properties involved with the activation of radiation and by studies showing conditioned responses and learned avoidance behavior. It is also evident that these responses vary in type and depend on a variety of mechanisms. The major responses reported in the literature are changes in food and water consumption, radiotaxis, avoidance of ionizing radiation, behavioral arousal, and neural responses in the olfactory and visual receptors.

We found no linear pattern for changes in food and water consumption, radiotaxis, behavioral arousal, or olfactory responses to increasing doses of ionizing radiation. The search for a pattern was greatly complicated by the fact that these studies were spread over many decades. Over this time, there have been advances in technology and large changes in the way that radiation doses are reported, resulting in a large non-uniformity in how experiments were conducted, and results were reported. Furthermore, there were a large variety of animal models used in the studies. These inconsistencies make generalizations of dose-response relationships difficult.

Broadly speaking, photoreceptors, and to a lesser extent chemoreceptors, are implicated in many of the sensory effects of low dose radiation. However, future research will need to more closely examine whether there is a phosphene effect of some kind in the visual system or whether there truly are receptors within the visual system that respond very specifically to radiation *via* increased excitation or increased amount of generated action potentials. With high radiation doses delivered to the whole body, there is a pattern of avoidance identified in response to ionizing radiation and in rats this response can be mitigated by surgical excision of a segment of one or more splanchnic nerves.

It is clear that the retina and photoreceptors mediate the visual response of X-ray energies, while extraretinal processes may be important in other forms of ionizing radiation generated phosphenes. Fluorescence has often been suggested as a mechanism for visual stimulation, but there is little evidence for this at X-ray energies. The response to low dose X-rays has been shown to have properties much like light perception in many species including the production of light ERGs and neural spiking behavior. There have also been interactions observed between light reception and X-ray reception. Notably, detection often, but not always, requires a dark-adapted photoreceptor and in mammals the rod photoreceptor is implicated. Paradoxically, it has been reported that X-rays do not cause photobleaching of rhodopsin or “visual purple.”

In olfactory and chemosensory responses much evidence suggests that the olfactory bulb is the site of action in rats and antennae, or other chemosensory organs are the site of action in invertebrates. Removal of these organs eliminates these sensory responses, and it is possible to observe the neural response electrophysiologically.

Taken together the evidence suggests that brief pulses of low dose X-rays in the range of 0.01–1 Gy per s can activate several classes of sensory receptors through some still poorly understood mechanism. As discussed above, our group believes that ROS production by X-rays and activation of receptor proteins are the most likely mechanism of visual and olfactory response. Past studies have concluded that radiolysis of water predominates X-rays impact on biological systems leading to the generation of ROS (Zaider, 1988). Specifically, pH-neutral aqueous solutions produce ~42 nM diatomic hydrogens, ~60 nM hydrogen atoms, ~71 nM hydrogen peroxide, ~222 nM hydroxyl radicals, ~230 nM solvated electrons per Gy of energy deposited (Spinks and Woods, 1990). ROS are well-documented cellular messengers and at higher concentrations oxidative stressors that induce changes in cellular function.

With respect to the doses described in these experiments, one Gray is a substantial radiation dose, but is not significantly dangerous. For context, a human being, on average a 1 Gr whole-body dose would have about a 5% chance of inducing a lethal cancer. The LD50 level for a whole-body dose is about 3.5 Gy, but the risk of death below 3 Gy is effectively zero. For localized doses, 1 Gy to the skin would have no clinical effect, though at levels above about 2–5 Gy it is possible to have effects like transient erythema. However, the tolerance of radiation can vary by orders of magnitude between species. For example, nematodes exhibit a much higher tolerance for radiation than humans. Weidhaas studied the effect of irradiation on the vulva of *C. elegans*; the report found that there is no significant lethality for doses of up to 500 Gy, and with only a small change in the vulva phenotype at 100 Gy (Weidhaas et al., 2006). In 1960, Meyers reported that the dose required for complete sterilization of populations of various species of nematode varied from about 400 Gy to higher than 1,600 Gy. The paper “Oxidative stress pretreatment increases the X-radiation resistance of the nematode Caenorhabditis elegans” looked at the lethality of X-rays on c-elegans; their data shows an LD50 of between 300 and 400 Gy.

Based on these reports, we speculate that X-rays could be used in a way analogous to how visible light is used for optogenetics. Others have used the term “X-genetics” for the use of X-rays for neuromodulation, a nomenclature we will adopt here. As far as we are aware, Rachel Berry and Ge Wang were the first to use this term. The premise of X-genetics is to modulate neural activity by expressing receptor proteins in neurons to confer radiosensitivity to neurons. In other words, we propose that there is an array of metabotropic or ionotropic receptor proteins with neuromodulation potential similar to channelrhodopsin that could be activated by X-rays. The compelling evidence for direct activation of visual receptors by X-rays also suggests that scintillators or transduction of X-rays to light may be an unnecessary component for X-genetics. If ROS production is indeed the mediator for X-ray transduction, then particles or molecules that enhance ROS production may be a more suitable substrate for enhancing X-genetic efficiency.

## Author contributions

VM, JB, CR, DJ, and MB conceived the initial ideas and wrote the manuscript. All authors contributed to the article and approved the submitted version.

## Conflict of interest

The authors declare that the research was conducted in the absence of any commercial or financial relationships that could be construed as a potential conflict of interest.

## Publisher's note

All claims expressed in this article are solely those of the authors and do not necessarily represent those of their affiliated organizations, or those of the publisher, the editors and the reviewers. Any product that may be evaluated in this article, or claim that may be made by its manufacturer, is not guaranteed or endorsed by the publisher.

## Footnotes

^1^A phosphene is generally defined to be a sensation of light in the visual system that is produced by something other than light. So, strictly speaking, “X-ray phosphene” is an oxymoron.
